# Targeting DAD1 gene with CRISPR-Cas9 system transmucosally delivered by fluorinated polylysine nanoparticles for bladder cancer intravesical gene therapy

**DOI:** 10.7150/thno.88550

**Published:** 2024-01-01

**Authors:** Dongdong Tang, Yang Yan, Yangyang Li, Yuqing Li, Junqiang Tian, Li Yang, Hui Ding, Ghassan Bashir, Houhong Zhou, Qiuxia Ding, Ran Tao, Shaohua Zhang, Zhiping Wang, Song Wu

**Affiliations:** 1 Department of Urology, Lanzhou University Second Hospital, Lanzhou 730030, China.; 2 Department of Urology, The Third Affiliated Hospital of Shenzhen University (Luohu Hospital Group), Shenzhen University, Shenzhen 518000, China.; 3 Department of Urology, South China Hospital, Medical School, Shenzhen University, Shenzhen 518000, China.; 4 Department of Urology, The First Affiliated Hospital of Zhengzhou University, Zhengzhou, 450052, China.; 5 Songshan Lake Materials Laboratory, Dongguan, 523808, China.

**Keywords:** fluorinated polylysine nanoparticles, CRISPR-Cas9 system, DAD1, bladder cancer, intravesical therapy

## Abstract

**Background:** Intravesical chemotherapy is highly recommended after transurethral resection of bladder tumor for patients with bladder cancer (BCa). However, this localized adjuvant therapy has drawbacks of causing indiscriminate damage and inability to penetrate bladder mucosal.

**Methods:** Fluorinated polylysine micelles (PLLF) were synthesized by reacting polylysine (PLL) with heptafluorobutyrate anhydride. Anti-apoptotic gene defender against cell death 1 (DAD1) was selected by different gene expression analysis between BCa patients and healthy individuals and identified by several biological function assays. The gene transfection ability of PLLF was verified by multiple* in vitro* and *in vivo* assays. The therapeutic efficiency of PLLF nanoparticles (NPs) targeting DAD1 were confirmed by intravesical administration using an orthotopic BCa mouse model.

**Results:** Decorated with fluorinated chains, PLL can self-assemble to form NPs and condense plasmids with excellent gene transfection efficiency *in vitro*. Loading with the CRISPR-Cas9 system designed to target DAD1 (Cas9-sgDAD1), PLLF/Cas9-sgDAD1 NPs strongly inhibited the expression of DAD1 in BCa cells and induced BCa cell apoptosis through the MAPK signaling pathway. Furthermore, intravesical administration of PLLF/Cas9-sgDAD1 NPs resulted in significant therapeutic outcomes without systemic toxicity* in vivo*.

**Conclusion:** The synthetized PLLF can transmucosally deliver the CRISPR-Cas9 system into orthotopic BCa tissues to improve intravesical instillation therapy for BCa. This work presents a new strategy for targeting DAD1 gene in the intravesical therapy for BCa with high potential for clinical applications.

## Introduction

Bladder cancer (BCa) is one of the most prevalent malignant tumors in the urinary system, with the male incidence ranking fourth worldwide 1, 2. Non-muscle-invasive BCa (NMIBC) is one of the most significant type of BCa, with an initial diagnostic rate of 75% 1. The standard treatment strategies for NMIBC include transurethral resection of bladder tumors and subsequent intravesical instillation of chemotherapy drugs such as mitomycin C, pirarubicin and Bacillus Calmette-Guérin (BCG), which can effectively reduce the recurrence rate 3. However, the 5-year recurrence and progression rates of NMIBC remain high at 50-70% and 10-30%, respectively 4, 5. The limitations of intravesical administration include unsatisfactory drug penetration through the urinary epithelial barrier, serious side effects and short retention time in the bladder 6. Therefore, novel and effective intravesical instillation therapies are urgently required for BCa treatment.

Gene therapy has been applied to treat several diseases, such as cancer, viral infections, and hereditary diseases 7. Compared to traditional chemotherapy for BCa, gene therapy has a huge advantage in overcoming the defects associated with chemotherapy, such as chronic/acute toxicity in multiple organs, drug resistance, and other drawbacks 8. However, the main challenge in gene therapy is the lack of safe and effective gene delivery vectors *in vitro* and *in vivo*. The clustered regularly interspaced short palindromic repeats (CRISPR)/CRISPR-associated protein 9 (Cas9) system has recently been developed for therapeutic genome editing and has gained widespread attention owing to its incredible genome editing performance 9, 10. Generally, the CRISPR-Cas9 system consists of a single-guide RNA (sgRNA) that matches the target DNA fragment and a Cas9 protein with a nuclease function 11. The CRISPR-Cas9 system has demonstrated enormous potential for treating cancer and genetic diseases such as transthyretin amyloidosis 12, β-hemoglobinopathies 13, nervous system disorders 14, and human immunodeficiency syndrome 15. Meanwhile, delivery strategies for CRISPR-Cas9 system using viral vectors raise safety concerns regarding immunogenicity and insertional mutagenesis 16-19. As attractive candidates, nonviral vectors, cationic polymers for example have been applied to the delivery of gene therapeutic tools because of several outstanding features, such as ease of synthesis, application versatility, and improved safety profiles 20, 21. After condensation into NPs by cationic polymer via electrostatic interactions, nucleic acid drugs are protected from enzymatic degradation and readily delivered across the cell membrane in a cytocompatible manner 22-25. In addition, the fluorination modification of these polymers, which generates a unique “fluorous effect,” remarkably improves the stability of the complex and facilitates endocytosis, lysosome escape, and intracellular nucleic acid release 26-29. It has been well documented that the gene transfection ability of cationic polymer can be sharply optimized after the decoration of fluoroalkyl chains 30-32. Meanwhile, fluorous ligands such as fluoroalkyls are beneficial for improving serum stability, cellular uptake, endosomal escape, and intracellular nucleic acid release, endowing the fluorinated polymers with a specific fluorous effect in gene delivery 33. Polymers, including polyethyleneimine (PEI), dendrimers, and polypeptides decorated with fluoroalkyl chains, can achieve excellent transfection efficiency and impressive serum tolerance at extremely low nitrogen-to-phosphorus (N/P) ratios 34. PLL (ε-Poly-L-lysine), an antibacterial agent approved by FDA, is a naturally occurring peptide and biodegradable polymer with a molecular weight of approximately 4000 Da 35. Inspired by our previous studies that fluorinated polyethyleneimine and chitosan possess high molecular weight exhibited excellent cross-membrane, transmucosal, and intratumoral penetration abilities for BCa treatment with negligible side effects, PLL may obtain these transmucosal capacities with an admirable transfection efficacy when decorated with fluoroalkyl chains 36-39. Since fluoroalkyl substances are both hydrophobic and lipophobic and prefer to associate with other fluorous compounds owing to a fluorophilic effect, fluorinated PLL may self-assemble into non-compact micelles with excellent capacity to efficiently condense nucleic acids into stable complexes 27. Report showed fluorinated PLL can be rapidly metabolized in the liver *in vivo* with robust gene transfection performance *in vitro*, indicating its favorable biocompatibility 40. Hence, it is well worth to investigate the low molecular weight of PLL for the *in vivo* gene therapy of cancer rather than the relatively high cytotoxicity of fluorinated polymers with high molecular weight.

**Figure 1:**
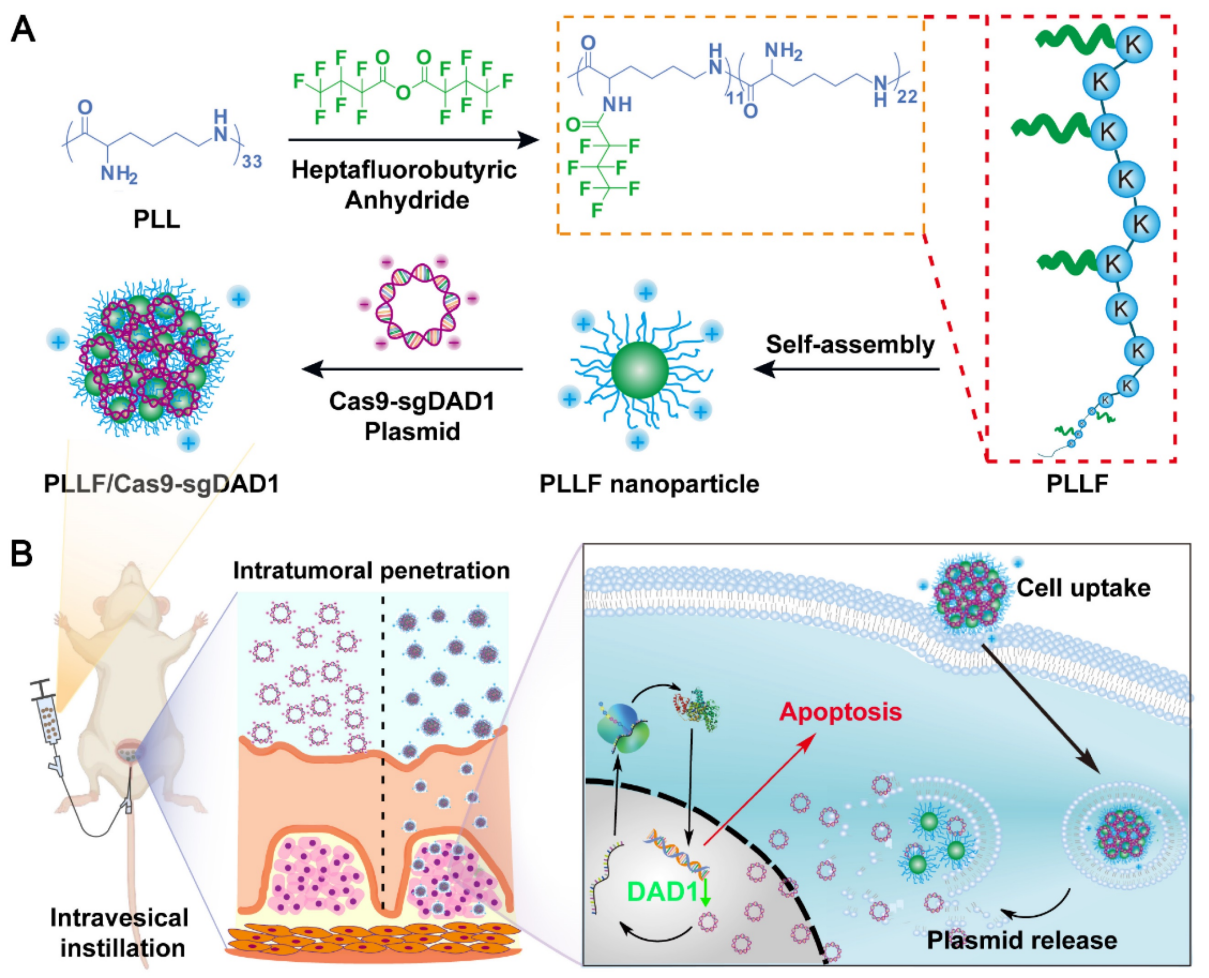
Schematic representation showing A) the preparation of PLLF/Cas9-sgDAD1 NPs and B) intravesical administration of PLLF/Cas9-sgDAD1 NPs using an orthotopic bladder tumor mouse model.

Here, we report a transmucosal delivery system based on PLLF NPs capable of efficiently delivering CRISPR-Cas9 system into orthotopic bladder tumors via intravesical instillation (**Figure 1**). PLL was decorated with fluoroalkyl chains to form PLLF micelles, which self-assembled into non-compact NPs in aqueous solutions. Subsequently, the CRISPR-Cas9 plasmid targeting a glycosylation-associated gene DAD1, a new promising target for BCa therapy acquired through public data analyses and experimental verifications, was condensed by PLLF via electrostatic interactions to obtain stable NPs (denoted as PLLF/Cas9-sgDAD1) (**Figure 1A**) 41, 42. With high cellular and mucosal penetrability, PLLF/Cas9-sgDAD1 NPs significantly downregulated the mRNA and protein levels of DAD1 in 5637 cells, resulting in cancer cell apoptosis. Intravesical administration of PLLF/Cas9-sgDAD1 NPs achieved a desired therapeutic outcome in an orthotopic bladder tumor mouse model and exhibited good biocompatibility (**Figure 1B**). Overall, this transmucosal delivery system can innovate future BCa gene therapies.

## Materials and methods

### Materials

PLL (ε-Poly-L-lysine) and heptafluorobutyric anhydride were purchased from Macklin Biochemical Co., Ltd. (Shanghai, China). Cy5.5 NHS was provided by APExBIO Technology LLC Co., Ltd. (Texas, USA). Poly (ethylene imine) (PEI 25K) was purchased from Sigma-Aldrich (St. Louis, Ltd (Missouri, USA). TransExcellent^TM^ DNA gene transfection reagent (TE^TM^) was bought from Cenji Biotech. Inc Co (Shanghai, China). Cell counting kit (cck-8) and Annexin V-FITC/PI cell apoptosis detection kits were supplied by TransGen Biotech Co., Ltd. (Beijing, China). The MGHU3 cell line was provided by Leibniz-Institut Deutsche Sammlung von Mikroorganismen und Zellkulturen GmbH (DSMZ). The 293T, SW780, J82, T24, RT4, and 5637 cell lines were purchased from American Type Culture Collection (ATCC). The immortalized human bladder epithelial cell line was provided by Nanjing Cobioer Co., Ltd. (Nanjing, China). The Cell/Tissue Total RNA Kit, Hifair™ II 1st Strand complementary DNA (cDNA) Synthesis Super Mix for qPCR, and Hieff UNICON^®^ Universal Blue qPCR SYBR Green Master Mix were purchased from Yeasen Biotechnology Co., Ltd. (Shanghai, China). The primary antibodies used in this work were as follows: DAD1 (Abclonal, A14723, USA), ERK (HUABIO, SA43-03), JNK (HUABIO, SA43-06), P38 (HUABIO, SR43-04, China), phospho-ERK (HUABIO, ER64553, China), phospho-JNK (HUABIO, ST500, China), and phospho-P38 (HUABIO, ER2001-52, China).

### Acquisition of key genes for glycosylation modification

Patients with complete sequencing data (FPKM) and prognosis information were filtered from the BCa dataset downloaded from The Cancer Genome Atlas Genomic Data Commons (TCGA GDC) for analysis. These eligible data were homogenized using the “limma” package, and gene annotation was accomplished according to the grch38.p13 from the human genome browser (http://asia.ensembl.org/index.html). The DEGs between BCa tissues and normal bladder tissues were obtained with the screening conditions of FDR < 0.05 and | Log_2_ (fold change) | > 1. A glycosylation modification-associated gene set containing 74 genes was downloaded from GSEA website (https://www.gsea-msigdb.org/gsea/index.jsp). The differential genes associated with glycosylation modifications were acquired through the intersection of the obtained DEGs and the glycosylation modification-associated gene set. Univariate regression analysis was used, combined with the patient survival time information, to determine the key glycosylation genes significantly related to the prognosis of patients with BCa.

### Cell culture

Human embryonic kidney epithelial cell line 293T, human bladder epithelial cell line SV-HUC-1, and human BCa cell lines, including T24, 5637, SW780, J82, RT4, and MGHU3, were cultured in Roswell Park Memorial Institute (RPMI) 1640 medium containing 10% fresh fetal bovine serum (Gibco) at 37 ℃ with 5% CO_2_. Additionally, 100 U/mL of penicillin and 100 μg/mL of streptomycin were added to the culture medium before use. The stable luciferase-expressing 5637 cell line (5637^luc^) was acquired by transfection with a luciferase-expressing plasmid (Ubi-MCS-firefly Luciferase-IRES-Puromycin) and then selected with 3 g/mL of puromycin.

### Knockdown DAD1 by siRNAs

siRNA targeting DAD1 was purchased from GenePharma (Shanghai, China) and the sequences were listed as follows:

siRNA (nc), sense: 5'-UUCUCCGAACGUGUCACGUTT-3', antisense: 5'-ACGUGACACGUUCGGAGAATT-3'; siDAD1-1, sense: 5'-GCCUGAGAAUACAGAUCAATT-3', antisense: 5'-AUACAGCAGGUACGCGUCCTT-3'; siDAD1-2, sense: 5'-GGACGCGUACCUGCUGUAUTT-3', antisense: 5'-AAGUACUCUUCUAAGAACCTT-3'.

BCa 5637 cells (3×10^5^ cells/well) were seeded in a 6-well cell culture plate the day before transfection. 100 pmol of siRNA were transfected with 5637 tumor cells using 6 μL GP-transfect-Mate transfection reagent (GenePharma, Shanghai, China). After 48 h, the expression of DAD1 was examined by qPCR and WB. For cell viability evaluation, 5637 tumor cells transfected with siRNA were transferred into a 96-well cell culture plate. After incubation with 10 μL of cell counting kit-8 reagent and 90 μL of 1640 medium per well for 1 h, the absorbance at 450 nm was detected using a Spark^®^ Multimode Microplate Reader (TECAN, Switzerland) at different time points (0, 1, 2, and 3 days).

### RT-qPCR

Total cellular RNA was extracted using the Cell/Tissue Total RNA Kit, and the final RNA concentration was measured using a NANODROP ONE C instrument (Thermo Fisher, USA). Then, 1 μg of total RNA was reverse-transcribed to cDNA using Hifair™ II 1st Strand cDNA Synthesis Super Mix for qPCR. Next, Hieff UNICON^®^ Universal Blue qPCR SYBR Green Master Mix was used to amplify the cDNA samples on an ABI 7500 Real-Time PCR system (QS Dx, Thermo Fisher, USA). GAPDH served as the internal reference, and the relative expression of DAD1 was calculated using the of 2^-ΔΔCt^ method. The primer sequences for DAD1 and GAPDH were as follows:

Forward (DAD1): 5'-GATTCTAGAGCTAGCGATGTCGGCGTCGGTAG-3', Reverse (DAD1): 5'-TCGCGGCCGCGGATCTCAGCCAACAAAGTTCATGA-3', Forward (GAPDH): 5'-CATCATCCCTGCCTCTACTG-3', Reverse (GAPDH): 5'-GCCTGCTTCACCACCTTC-3'.

### Western Blotting

Cell and tissue samples were lysed using RIPA (Solarbio, R0100, China) containing a 1% complete Protease Inhibitor Cocktail (Roche, 11873580001, Switzerland). An additional sonication step was performed for the tissue samples using an Ultrasonic Cell Pulverizer (UP-250) to assist with total protein extraction. Total proteins were separated on a 12% SDS-PAGE gel and transferred to a polyvinylidene fluoride (PVDF) membrane (Millipore, USA). The membrane was blocked with 5% skim milk at room temperature for 2 h. After incubation with the corresponding primary antibody at 4 ℃ overnight and horseradish peroxidase-labeled secondary antibody (Jackson ImmunoResearch, Pennsylvania, USA) at room temperature for 1 h, the membrane was reacted with an enhanced chemiluminescence reagent (Millipore, USA) and imaged with an automatic chemiluminescence imaging system (GelView 6000 Pro, Guangzhou, China).

### Immunohistochemistry (IHC)

IHC staining was performed on paraffin-embedded sections of xenograft bladder tumor tissues and human BCa tissue microarray. The microarray named HBlaU108Su01(Outdo Biotech, Shanghai, China) included 40 pairs of adjacent normal tissues, BCa tissues, and 28 other BCa tissue samples. Clinical and follow-up information was also provided. The sections were dewaxed, hydrated, fixed, and subjected to high-temperature antigen repair. Hydrogen peroxide (3%), bovine serum albumin (1%), and triton (0.1%) were used to remove endogenous catalase and block the permeability of the sections. The sections were incubated with the primary antibody at 4 ℃ overnight, biotin-conjugated secondary antibody, and streptavidin-horseradish peroxidase (HRP) at room temperature for 30 min. The sections were visualized using 3,3'-diaminobenzidine (DAB) and scanned using an Aperio ScanScope XT (Leica Microsystems, Germany). The expression of DAD1 in tissue microarray samples was divided into high and low expression groups according to staining intensity using ImageJ software.

### Construction of the CRISPR-Cas9-sgDAD1 plasmid

The genome sequence of the human DAD1 gene (gene ID: 1603) was obtained from the NCBI website, and the online website (https://www.atum.bio/catalog/vectors/grna-design) was used for the design of the DAD1 targeting sgRNA sequence. The sgRNA sequence was as follows: Forward: 5'-CACCGGTAACCGAACTGCAGCGCCC-3,' Backward: 5'-AAACGGGCGCTGCAGTTCGGTTACC-3'. After annealing, the sgRNA sequence was subcloned into the pLentiCRISPR-V2 plasmid using the *BsmB* I site by T4 DNA ligase (NEB, USA) to form the Cas9-sgDAD1 plasmid. Subsequently, 4 μL of the recombinant Cas9-sgDAD1 plasmid was transformed into 50 μL of competent bacterial cells (Stbl 3) purchased from Shenzhen KT Life Technology Co., Ltd. After the recombinant Cas9-sgDAD1 plasmids were extracted using the E.Z.N.A^®^ Endo-free Plasmid Mini Kit Ⅱ (Omega, USA) according to the manufacturer's protocol, they were identified by Sanger sequencing (RiboBio, Guangzhou, China) and stored at -20 ℃ for further use.

### Preparation and Characterization of PLLF/plasmids NPs

PLLF were synthesized according to a previously reported method 43. Briefly, 100 mg PLL, 160 μL of heptafluorobutyric anhydride (7F), and 120 μL triethylamine were dispersed in 4 mL absolute methanol. After reacting at room temperature for 48 h with stirring, the mixed solution was dialyzed against distilled water using a dialysis bag (molecular weight cutoff ~1000 Da). A well-established ninhydrin assay was used to measure the number of residual amine groups on PLLF. The resulting PLLF NPs were dried using a rotary evaporator (YRE-201D, Shanghai, China) and resuspended in distilled water at a final concentration of 2 mg/mL.

Various amounts of PLLF were mixed with 0.8 μg of CRISPR-Cas9 plasmid, where the N/P values were set at 0.5, 0.75, 1, 2, 4, and 8, respectively, and incubated at room temperature for 30 min. N denotes the number of residual primary amines on PLLF, and P represents the number of phosphate anions in the DNA chains. The PLLF/DNA NPs and free plasmid were run on 1% agarose gels at 100 V for 30 min.

The size distribution and zeta potential of the synthesized NPs, including PLLF and PLLF/CRISPR-Cas9 plasmid, were measured using Zetasizer Nano ZS (Zetasizer Nano ZS-90, Malvern, UK). The morphological features were characterized using a transmission electron microscope (TEM; JEM-1200 EX, Japan) at an accelerating voltage of 120 kV.

### Transfection of pEGFP plasmid

To evaluate the plasmid delivery efficiency of PLLF, BCa 5637 (3×10^4^ cells/well) or 293T cells (1×10^4^ cells/well) were seeded in a 24-well cell culture plate and then incubated with different formulations, including PLL/pEGFP (N/P = 4), PEI/pEGFP (as per vendor's protocol), PLLF/pEGFP (N/P = 4), TE^TM^/pEGFP (as per vendor's protocol) at a plasmid dose of 0.8 μg per well. After a 6-hour co-culture, the culture medium was replaced with a fresh RPMI 1640 complete culture medium. EGFP expression was confirmed using an inverted fluorescence microscope (Zeiss Axio Observer, Germany) or quantitatively analyzed by flow cytometry (BD FACSCalibur, USA) at the time point of 24 h.

### Investigation of PLLF penetration in 3D cell spheroids

For the preparation of 3D tumor cell spheroids, BCa cells 5637 were seeded in a 96-well plate with an ultra-low attachment surface at a concentration of 1.2×10^4^ cells/well. The formed tumor cell spheroids were incubated with PLL/pEGFP (N/P = 4), PEI/pEGFP (as per vendor's protocol), TE^TM^/pEGFP (as per vendor's protocol), and PLLF/pEGFP NPs (N/P = 4) at a plasmid dose of 1.2 μg per well for 6 h, and the culture medium was then replaced with a fresh RPMI 1640 complete culture medium. After another 24-hour culture, EGFP expression in the tumor spheroids was detected using a confocal laser scanning microscope (CLSM, Zeiss LSM800 with Airyscan, Germany).

### Ussing Chamber Assay

The mucosal layer separated from the excised mouse bladder was washed with PBS and then settled in the Ussing chamber device with a constant mucosal area of 0.1 cm^2^. The urothelial mucosa was fixed to the feeding tank of the chamber. The feeding tank was filled with Tyrode's solution (Solarbio, Beijing, China) containing Cy5.5-labeled PLL/pEGFP or PLLF/pEGFP, whereas the reception tank was filled with fresh Tyrode's solution. The Ussing chamber was maintained at 37 ℃ and supplied with O_2_/CO_2_ (95/5%). 100 μL Tyrode solution in the reception tank was pipetted out after 15, 30, and 60 min for Cy5.5 signal detection using a Spark^®^ Multimode Microplate Reader (TECAN, Switzerland). The resulting value for the PLL/pEGFP group after 15 min was used as the control to calculate the relative penetration efficiency.

### Synthesis of Cy5.5-labeled PLL/pEGFP and PLLF/pEGFP

Cy5.5 NHS ester was dissolved in dimethyl sulfoxide (DMSO) to a concentration of 5 mg/mL. To label PLL/pEGFP or PLLF/ pEGFP NPs with Cy5.5, and 5 μL of Cy5.5 NHS solution was added to 5 mg of PLL/pEGFP and PLLF/pEGFP NPs in a final volume of 1 mL, respectively, and the reaction continued under stirring for 24 h at room temperature in a dark environment. Subsequently, a dialysis bag (~1000 Da; Biosharp, USA) was used to dialyze the reaction mixture against pure water. After the lyophilization of the dialysis product, Cy5.5-labeled PLL/pEGFP and PLLF/pEGFP NPs were obtained and stored at 4 ℃ until further use.

For the frozen section assay, female BALB/c mice (6-8 weeks old) with orthotopic bladder tumors were anesthetized with pentobarbital sodium (50 mg/kg) and intravesically instilled with Cy5.5-labeled PLL/pEGFP (N/P = 4) or PLLF/pEGFP (N/P = 4) at a plasmid dose of 10 μg. The bladders were collected after 2 h and embedded in Tissue-Tek optimal cutting temperature compound (SAKURA, USA). A freezing microtome (HM525 NX, Thermo Fisher Scientific, USA) was used to prepare the frozen sections. The distributions of Cy5.5-labeled PLL/pEGFP and PLLF/pEGFP were detected using CLSM (Zeiss LSM800 with Airyscan, Germany).

### Cytotoxicity assessment

5637 or SV-HUC-1 cells were inoculated evenly into a 96-well cell culture plate at a density of 2000 cells per well and incubated in RPMI 1640 complete medium at 37 ℃ with 5% CO_2_ for 24 h. The mixture of the Cas9-sgDAD1 plasmid, PLL, PEI, PLLF, and TE^TM^ was prepared as described above. The plasmid dose was 0.1 μg per well. The cell viability was calculated by cell counting kit-8 reagent.

For flow cytometry to detect apoptosis, 5637 cells were collected after treatment for 48 h, washed twice with cold PBS, and resuspended in 100 μL of cold 1×Annexin V binding buffer. After incubation with Annexin V-FITC and PI for 15 min at room temperature, the samples were analyzed using FCM cytometry (BD FACS Calibu, USA). To evaluate the cytotoxicity of the 3D tumor spheroids, the formed tumor cell spheroids were incubated with PLL/Cas9-sgDAD1 (N/P = 4), PEI/ Cas9-sgDAD1 (as per vendor's protocol), TE^TM^/Cas9-sgDAD1 (as per vendor's protocol), and PLLF/ Cas9-sgDAD1 NPs (N/P = 4) at a plasmid dose of 1.2 μg per well for 6 h, and the culture medium was then replaced with a fresh RPMI 1640 complete culture medium. they were stained with Hoechst33342 and PI at room temperature for 30 min after incubation with the different formulations for 48 h. Fluorescent images were captured using CLSM (Zeiss LSM800 with Airyscan, Germany).

### Transcriptome sequencing analysis

After DAD1 expression was repressed by the application of PLLF/Cas9-sgDAD1 in BCa 5637 cells, total RNA was extracted and quantified as described above. Cells treated with PLLF served as controls. The VAHTS^®^ Universal V6 RNA-seq Library Prep Kit (Vazyme, Nanjing, China) was used to prepare the cDNA library according to the manufacturer's protocol. Transcriptome sequencing was performed by Novogene Co., Ltd. (Beijing, China). |Log_2_(fold change) | > 1 and *P* < 0.05 were set as the criteria to obtain the DEGs. The analysis of the sequencing data and enrichment of differential gene functions were completed by the “clusterProfiler” R package.

### Animals

BALB/c nude mice (6 weeks old, female) were purchased from GemPharmatech Co., Ltd. (Guangdong, China) and raised in distilled water and sterilized food under specific pathogen-free conditions. All animal experiments were conducted in accordance with the National Guidelines for the Care and Use of Experimental Animals and were approved by the Experimental Animal Ethics Committee of Shenzhen Zhongxun Precision Medicine Research Institute.

### PLLF/Cas9-sgDAD1 intravesical instillation therapy of orthotopic bladder cancer

The preparation of the inoculated tumor cells was done by digesting BCa stable luciferase-expressing 5637 cells (5637^Luc^) with trypsin and resuspended in serum-free 1640 medium solution containing 20% Matrigel (BD Biosciences). The cell suspension was adjusted to 2×10^7^ cells/mL and stored in an ice-water mixture until use. Then, 25 μL of cell solution (5×10^5^ cells per mouse) was inoculated into the bladder wall after the mice were anesthetized with 1% pentobarbital sodium (50 mg/kg) to establish an orthotopic BCa mouse model. Before the bioluminescence imaging of bladder tumors *in vivo*, tumor-bearing mice were intraperitoneally injected with 10 mg/kg d-luciferin potassium salt (Thermo Fisher Scientific). According to the fluorescence intensity examined by IVIS Lumina system (Caliper Life Sciences, Massachusetts, USA) 3 days after inoculation, these tumor-bearing mice were randomly divided into three groups (5 mice in each group) for the intravesical administration of different formulations, including 100 μL of PBS, PLL/Cas9-sgDAD1 (N/P = 4), and PLLF/Cas9-sgDAD1 (N/P = 4) at a plasmid dose of 10 μg per mouse using a closed IV catheter system (0.7 mm×19 mm, BD Intima II). After instillation, the urethra was clamped with a vascular clip to prevent overflow of the instillation solution, and the treatment was sustained for 1 h. Intravesical administration was repeated four times on days 5, 8, 11, and 14. Orthotopic tumor growth was imaged again on day 17 using the IVIS Lumina system. Subsequently, all mice were euthanized, and the bladders were immediately resected for imaging and weighing. Tumor-bearing bladders were subjected to the analyses of hematoxylin-eosin (H&E) staining, Ki67 IHC, and terminal deoxynucleotidyl transferase dUTP nick end labeling (TUNEL). In addition, the major organs, including the heart, liver, spleen, lungs, and kidneys, were excised for H&E staining. To determine DAD1 expression in orthotopic tumors, western blotting and IHC analyses were performed as described above.

### Statistical Analysis

All statistical analyses and figures were performed using GraphPad Prism 8.0 (GraphPad Software, Inc., La Jolla, CA, USA) or R software (version R-4.0.2). All data are expressed as mean ± standard deviation (SD). Statistical analyses were performed using a standard two-tailed Student's *t*-test or one-way analysis of variance (ANOVA). Survival curves were displayed using Kaplan-Meier curves. The significance levels were defined as *P* < 0.05 (*), *P* < 0.01 (**), and *P* < 0.001 (***).

## Results and Discussions

### Identification of DAD1 as a target for BCa treatment

**Figure 2:**
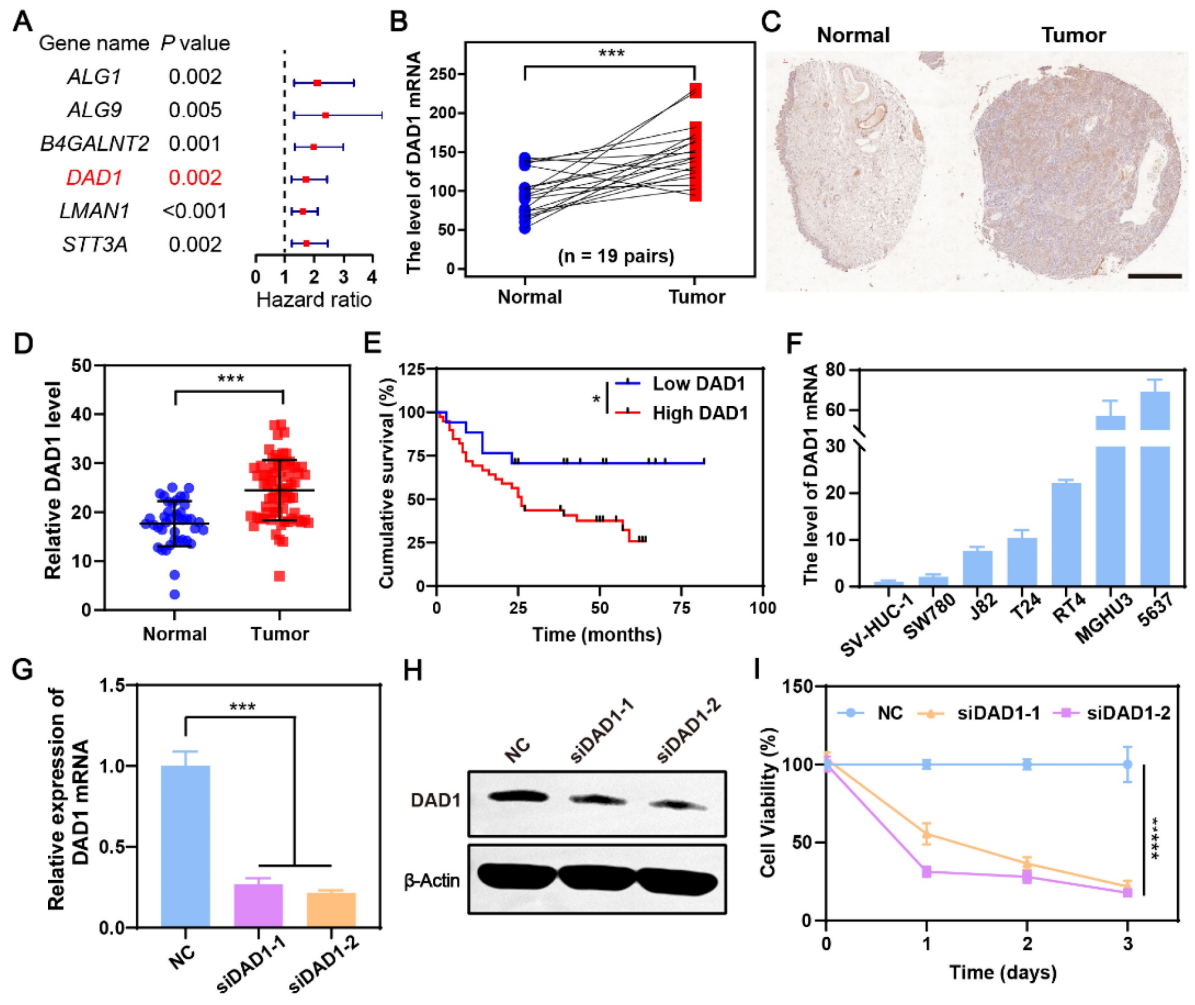
** DAD1 is a promising target for BCa treatment. A)** The filtrated glycosylation modification genes associated with the prognosis of BCa patients. **B)** The level of DAD1 mRNA in BCa tissues and paired normal tissues in TCGA database (N = 19 pairs). **C)** The representative immunohistochemistry images of DAD1 from tissue microarray HBlaU108Su01. Scale bar: 500 μm. **D)** Relative DAD1 level in tissue microarray HBlaU108Su01 (Normal = 40, Tumor = 68).** E)** Kaplan- Meier survival curve of DAD1 high expression and low expression groups in the tissue microarray HBlaU108Su01.** F)** The expression levels of DAD1 mRNA in normal bladder epithelial cell line SV-HUC-1 and BCa cell lines SW780, J82, T24, RT4, MGHU3, 5637 (N = 3). The expression levels of DAD1 **G)** mRNA and **H)** protein in 5637 cells with siRNA knockdown (N = 3).** I)** The effect of DAD1 knockdown by siRNA on cell viability of 5637 cells. Statistic significances were calculated by the Student's *t*-test two tailed. *P < 0.05, ***P < 0.001.

During the occurrence and development of BCa, glycosylation modifications in cancerous cells are dysregulated, which may result in novel gene targets for BCa treatment 44. Our previous work also found that dysregulation of glycometabolism, particularly dysregulation of glycosylation modifications, exists in BCa 45, 46. Referencing this, a differentially expressed gene set was obtained by comparing the gene expression of 19 normal bladder tissues and 411 BCa tissues in The Cancer Genome Atlas Genomic Data Commons (TCGA GDC), a well-known public database for cancer research. The resulting 8427 genes with a false discovery rate (FDR) < 0.05, and |Log2 (fold change) | >1 were then intersected with glycosylation modification genes downloaded from gene set enrichment analysis (GSEA), and 49 glycosylation modification differential genes were ultimately focused on** (Figure S1)**. Among them, six genes were significantly related to the prognosis of BCa patients with a hazard ratio>1, indicating that the higher the expression of the indicated genes, the worser the prognosis of BCa patients (**Figure 2A**). According to the result (**Figure 2B**), the anti-apoptotic gene DAD1 was expressed significantly higher in bladder tumors than in normal bladder tissues. Additionally, the expression of DAD1 exhibited a distinct negative correlation with patient prognosis **(Figure S2)**, suggesting its potential as a biomarker and therapeutic target in BCa. Tissue microarray HBlaU108Su01 analysis further validated the increased expression of DAD1 in BCa tissues (**Figure 2C-D** and **Figure S3**). The cumulative survival analysis of patients corresponding to the tissue microarray shown in **Figure 2E** also confirmed the importance of DAD1 in prognosis prediction. Subsequently, DAD1 mRNA expression was examined in several BCa cell lines, including T24, 5637, SW780, J82, RT4, and MGHU3, and in the normal bladder epithelial cell line, SV-HUC-1. Using SV-HUC-1 as the control, increased expression of DAD1 mRNA was observed in BCa cell lines, particularly in 5637 cells (around 70 folds) (**Figure 2F**), which were chosen to investigate the anti-apoptotic effectiveness of DAD1. Two small interfering RNAs (siRNAs) targeting different regions of the DAD1 mRNA were designed to suppress DAD1 expression. After siRNA treatment, both mRNA and protein levels in 5637 cells significantly decreased, as shown in **Figure 2G**-**H**, respectively. The viability of the DAD1-restrained 5637 cells was significantly impaired compared to that of the negative control (**Figure 2I**). The results of the wound-healing assay also demonstrated that the knockdown of DAD1 hindered the migratory ability of 5637 cells **(Figure S4)**. Previous studies have reported that the primary function of DAD1 is to negatively regulate programmed cell death and constitute the main component of oligosaccharyltransferase (OST) in the N-linked glycosylation of proteins 47. The increased expression of DAD1 in hepatocellular carcinoma, colon cancer, small intestinal carcinoid, chronic lymphocytic leukemia, and prostate cancer indicates its great potential as a tumor treatment target of DAD1 41. This work firstly verified the increased expression and anti-apoptotic function of DAD1 in BCa cells. Based on the results, DAD1 gene can be proposed as a novel gene target for BCa gene therapy.

### Preparation and characterization of PLLF/CRISPR-Cas9 NPs

**Figure 3:**
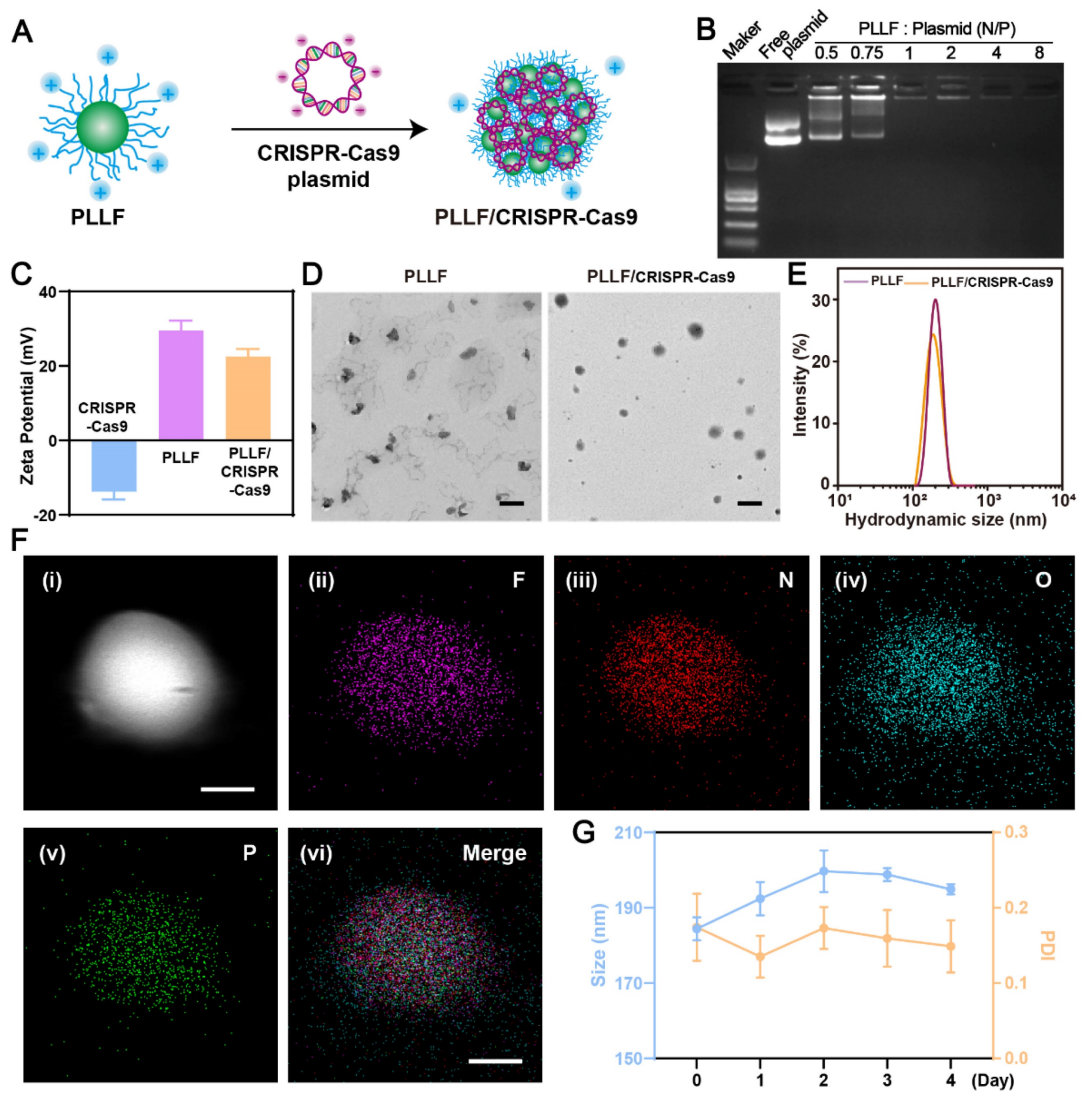
** Preparation and characterization of PLLF/CRISPR-Cas9 system. A)** Schematic representation showing the synthetic process of PLLF/CRISPR-Cas9. **B)** Agarose gel electrophoresis of free plasmid and PLLF/pEGFP at different N/P ratios. **C)** Zeta potentials of CRISPR-Cas9 plasmid, PLLF and PLLF/CRISPR-Cas9 plasmid. **D)** TEM images of PLLF and PLLF/CRISPR-Cas9 NPs. Scale bars: 200 nm. **E)** The hydrodynamic size distributions of PLLF and PLLF/CRISPR-Cas9 NPs. **F)** High-angle annular dark field image of a representative PLLF/CRISPR-Cas9 nanoparticle (i) and the corresponding elemental mapping images of F (ii), N (iii), O (iv), and P (v). Scale bars: 50 nm. **G)** Stability analysis of PLLF/CRISPR-Cas9 NPs kept at room temperature as measured by particle size and PDI at different time intervals.

To fabricate well-designed PLLF/CRISPR-Cas9 NPs, PLLF was first synthesized by reacting PLL with heptafluorobutyrate anhydride and the average number of fluoroalkyl chains conjugated on each PLL was about 11 according to the ninhydrin assay. ^1^H nuclear magnetic resonance (NMR) and Gel permeation chromatography (GPC) were carried out to verify the chemical structure of PLLF. The results of ^1^H NMR spectra (**Figure S5**) demonstrated the decoration of fluorinated chains on PLL. Number-average molecular weight (Mn) of PLL and PLLF were found to be 4128 and 6402, and the weight-average molecular weight (Mw) of PLL and PLLF were turned to be 4808 and 8154 respectively. Thus the average number of fluoroalkyl chains conjugated on each PLL was calculated to be 11.5 based on the value variation of Mn, which is consistent with the ninhydrin assay. The obtained PLLF immediately self-assembled into non-compact micelles via a well-established film dispersion method because of the hydrophilic PLL and hydrophobic 7F chains (**Figure 1A**). As shown in **Figure 3A**, the non-compact PLLF micelles turned to be more compact after the adsorption of large CRISPR-Cas9 plasmids through electrostatic interactions. The optimal binding conditions were explored by binding various amounts of PLLF micelles to the given quantity of plasmids (0.8 μg), in which the PLLF to plasmid (N/P) ratios were 0.5, 0.75, 1, 2, 4, and 8, respectively. Agarose gel electrophoresis (**Figure 3B**) indicated that the plasmids were completely retained at an N/P ratio above 2. Moreover, the PLLF/CRISPR-Cas9 NPs formed from different N/P values had a stable size distribution of approximately 200 nm, with a polymer dispersity index (PDI) lower than 0.3 (**Figure S6**). The PLLF/CRISPR-Cas9 NPs obtained at an N/P of 4 were used for subsequent analyses. Based on the results shown in** Figure 3C**, the zeta potential of PLLF/CRISPR-Cas9 (22.5 mV) was lower than that of PLLF (< 29.5 mV), owing to the binding of negatively charged CRISPR-Cas9 plasmids (**Figure 3C**). Transmission electron microscopy (TEM) images demonstrated a more compact structure and uniform spherical appearance of PLLF/CRISPR-Cas9 compared to those of PLLF micelles (**Figure 3D**), which may be attributed to the compression of electrostatic interactions. The hydrodynamic size of PLLF/CRISPR-Cas9 was slightly smaller than that of PLLF, with peaks at 185 and 218 nm, respectively (**Figure 3E**), consistent with the TEM observations. To further examine the chemical components of PLLF/CRISPR-Cas9 NPs, energy-dispersive X-ray spectroscopy (EDX) was performed using a field-emission transmission electron microscope (FETEM). A high-angle annular dark-field (HAADF) image of a representative PLLF/CRISPR-Cas9 nanoparticle is shown in** Figure 3F** (i), and the corresponding key elemental mappings are shown in **Figure 3F** (ii-v, indicating F, N, O, and P, respectively**)**. In addition, the stability of the PLLF/CRISPR-Cas9 NPs was evaluated by repeatedly measuring the particle size and PDI at different time points. The results shown in **Figure 3G** confirm the sustainable stability of the PLLF/CRISPR-Cas9 NPs when kept at room temperature for at least four days. These results indicated that PLLF/CRISPR-Cas9 NPs were successfully fabricated with satisfactory storage stability for delivering therapeutic CRISPR-Cas9 plasmids.

### Evaluation of the plasmid transfection *in vitro* and mucosal penetrability *in vitro* and *in vivo* by PLLF

**Figure 4:**
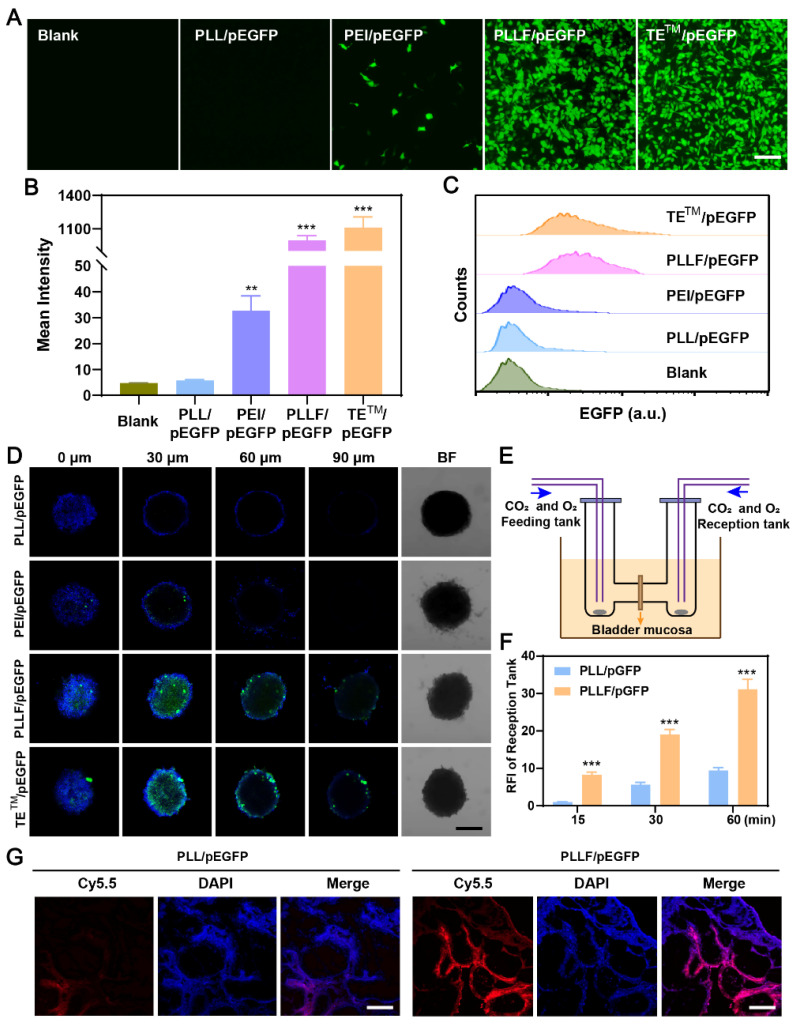
** Evaluation of the plasmid transfection *in vitro* and mucosal penetrability *in vitro* and *in vivo* by PLLF. A)** Fluorescence images of EGFP expression in 5637 cells after pEGFP plasmids were transfected with PLL, PEI, PLLF and TE^TM^. Scale bars: 100 μm. **B)** The corresponding mean intensity of EGFP in 5637 cells quantitated from three random views.** C)** Flow cytometry analysis of EGFP expression in 5637 cells after pEGFP plasmids were transfected with PLL, PEI, PLLF and TE^TM^. **D)** Confocal fluorescence images of 3D tumor spheroids generated by 5637 cells showing the cross-membrane penetrability of PLL/pEGFP, PEI/pEGFP, PLLF/pEGFP and TE^TM^/pEGFP. Scale bar: 200 μm. **E)** Schematic illustration of mini-Ussing chamber assay.** F)** Relative fluorescence intensity of reception tank with feeding of Cy5.5-labeled PLL/pEGFP and PLLF/pEGFP at 15, 30 and 60 min. Fluorescence intensity of reception tank with Cy5.5-labeled PLL/pEGFP feeding at 15 min was set as control. Data are presented as mean ± SD (N = 3).** G)** The assessment of transmucosal ability of Cy5.5-labeled PLL/pEGFP and PLLF/pEGFP *in vivo* through intravesical instillation. Red fluorescence indicates Cy5.5-labeled PLL or PLLF. Scale bars: 200 μm. Statistic significances were calculated by the Student's *t*-test two tailed. **P < 0.01. ***P < 0.001.

Fluorinated polymers exhibit excellent performance in cross-membrane penetration due to the hydrophobic and lipophobic behaviors of fluorocarbon chains, facilitating their applications in the delivery of therapeutic regents such as siRNA 32, 48, plasmid 26, and protein 49 for the treatment of various diseases. To visually verify the transmembrane transport effectiveness of PLLF, CRISPR-Cas9 plasmids were replaced with pEGFP plasmids capable of expressing enhanced green fluorescent protein (EGFP) during the synthesis process, and the yield products were denoted as PLLF/pEGFP. After co-culturing different formulations with 5637 cells for 24h and 48h, EGFP expression images showed that PLLF and the fluoropolymer DNA delivery material TE^TM^ (set as the positive control) had a comparable plasmid transfection efficacy, which was significantly better compared to that of PLL without fluorination modification and the common gene transfection reagent, PEI (**Figure 4A and Figure S7**). The corresponding fluorescence intensities are presented in **Figure 4B**. Similar fluorescence results were obtained from the human embryonic kidney cell line 293T (**Figure S8**). Flow cytometry analysis revealed more than 90% EGFP-positive 5637 cells in the PLLF/pEGFP and TE^TM^/pEGFP groups (**Figure 4C**). Different number of fluoroalkyl chains, including 5 (PLL5F), 9 (PLL9F), and 13 (PLL13F) were also modified on PLL, and the EGFP plasmid transfection results in 5637 cells at 48 h pronounced the best transfection performance of PLL11F than others (**Figure S9**). Subsequently, a three-dimensional (3D) tumor cell sphere model was established by culturing 5637 cells in a 96-well plate with an ultralow attachment surface to assess the cross-membrane penetration ability of PLLF/pEGFP. The results shown in **Figure 4D** indicate the high efficacy of PLLF/pEGFP and TE^TM^/pEGFP, owing to EGFP expression at the center of the cell spheres, in contrast to that of the PLL/pEGFP and PEI/pEGFP groups.

The bladder mucosa is a physiological barrier that preserves the bladder from pathogenic bacteria, viruses and exogenous toxins furthermore impairs the outcomes of intravesical instillation for bladder cancer. Fluorinated polymers with effective mucosal penetrability have been developed to carry chemical drugs via intravesical instillation. To evaluate the bladder mucosa penetrability of PLLF/pEGFP NPs, the mini-Ussing chamber model, a conventional *in vitro* system used to estimate the transport index, was employed, in which a piece of bladder mucosa from a mouse was stuck to separate the feeding and reception tanks, as shown in **Figure 4E** 37, 50. Equal amounts of Cy5.5-labeled PLL/pEGFP or PLLF/pEGFP NPs were added into the feeding tank, and the penetrated NPs in the reception tank were detected after 15, 30, and 60 min. PLLF/pEGFP NPs exhibited better bladder mucosa penetrability than PLL/pEGFP NPs (**Figure 4F**), indicating the importance of fluoroalkyl chains modification for penetration. PLL has minimal gene transfection ability owing to its low molecular weight and lack of pH buffering capacity. After being decorated with fluoroalkyl chains, PLL had significantly improved gene transfection efficacy and mucosal penetrability owing to the “fluorous effect,” which was consistent with a previous report 51. Collectively, these results suggest that the fabricated PLLF/CRISPR-Cas9 system has excellent cell and mucosal penetrability, promoting its application in BCa gene therapy via intravesical instillation.

### *In vitro* investigation of PLLF/Cas9-sgDAD1 NPs on bladder tumor inhibition

**Figure 5:**
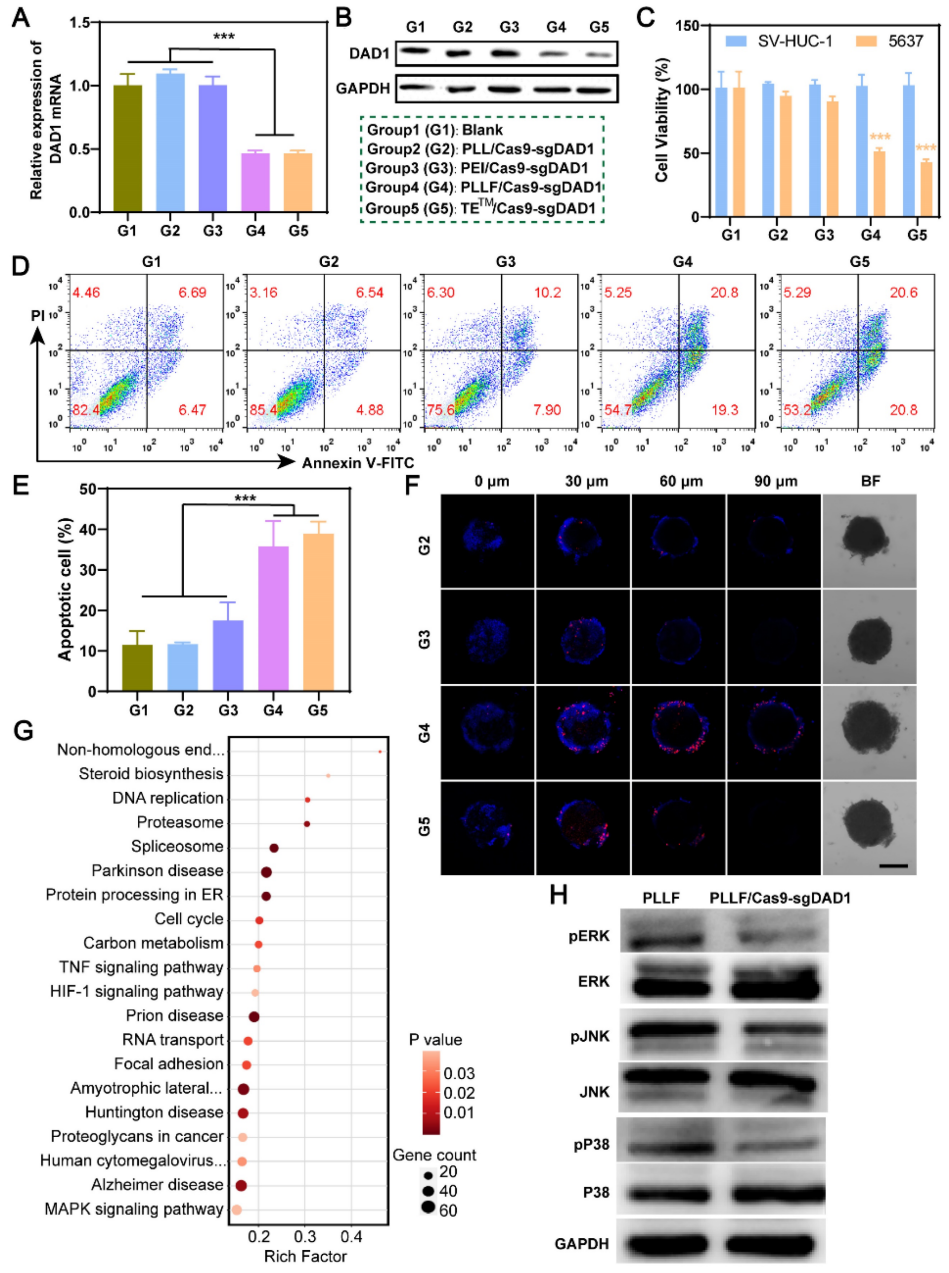
***In vitro* investigation of PLLF/Cas9-sgDAD1 NPs on bladder tumor inhibition.** The alterations of DAD1 mRNA **A)** and protein** B)** in 5637 cells after different treatments including group 1 (G1): Blank, group 2 (G2): PLL/Cas9-sgDAD1, group 3 (G3): PEI/ Cas9-sgDAD1, group 4 (G4): PLLF/ Cas9-sgDAD1 and group 5 (G5): TE^TM^/ Cas9-sgDAD1. **C)** The cell toxicity of SV-HUC-1 and 5637 cells with G1-G5 treatments. **D)** Flow cytometry analysis of 5637 cell apoptosis with G1-G5 treatments.** E)** The corresponding percentages of apoptotic cells. Data are represented as Mean ± SD (N = 3). **F)** Confocal fluorescence images of 3D tumor spheroids formed by 5637 cells showing the dead cells (red) after G2-G5 treatments. Scale bar: 200 μm.** G)** Pathway enrichment analysis of 33 DEGs between control and PLLF/Cas9-sgDAD1 treated 5637 cells. **H**) Western blotting assay showing the expression of ERK, P38, JNK, pERK, pP38 and pJNK in PLLF and PLLF/Cas9-sgDAD1 treated 5637 cells. ***P < 0.001.

Based on these results, the PLLF/CRISPR-Cas9 system targeting DAD1 is expected to be advantageous for effective BCa treatment via intravesical instillation. Therefore, a designed DNA segment was first inserted into the CRISPR-Cas9 plasmid to express a single guide RNA (sgRNA), which led to the CRISPR-Cas9 protein cleaving the double-stranded DAD1 gene in the genome. The detailed profile of the resulting CRISPR-Cas9 plasmid (Cas9-sgDAD1) is shown in **Figure S10**. To verify the tumor-inhibitory ability of PLLF/Cas9-sgDAD1 NPs, BCa 5637 cells were incubated with different formulations, including group 1 (G1): Blank, Group 2 (G2): PLL/Cas9-sgDAD1, group 3 (G3): PEI/Cas9-sgDAD1, group 4 (G4): PLLF/Cas9-sgDAD1, and positive control group 5 (G5): TE^TM^/Cas9-sgDAD1. Compared to G1, both the mRNA and protein levels of DAD1 in 5637 cells were downregulated in G4 and G5 but not in G2 and G3 (**Figure 5A-B**). These results demonstrated the efficient suppression of DAD1 by the Cas9-sgDAD1 plasmid when delivered using an appropriate method. Corresponding to the decrease in DAD1 expression, the viability of 5637 cells was also significantly reduced in the G4 and G5 groups (**Figure 5C**). However, PLLF/Cas9-sgDAD1 NPs showed no apparent toxicity in the normal bladder cell line SV-HUC-1 (**Figure 5C**), which may be attributed to the minimal expression of DAD1. This uneven cell toxicity indicated the specific tumor eradication ability of PLLF/Cas9-sgDAD1 NPs. Quantitative detection of apoptotic 5637 cells through flow cytometry analysis further demonstrated superior tumor destruction ability in G4 and G5, where tumor cells with positive apoptotic signals reached ~40%, in contrast to approximately 12% of apoptotic cells in G1 and G2 (**Figure 5D-E**). In addition, 3D tumor spheroids generated from 5637 cells were treated using the formulations mentioned above to evaluate the penetrable tumor lethality of PLLF/Cas9-sgDAD1 NPs. After incubation for 48 h, tumor spheroids and dead cells were stained with 2-(4-Amidinophenyl)-6-indolecarbamidine dihydrochloride (DAPI; blue) and propidium iodide (PI; red). As shown in the fluorescence images in **Figure 5F**, enhanced red fluorescence was observed in the interior of the tumor spheroids in G4 and G5, compared to that in G2 and G3, confirming the desired performance of PLLF/Cas9-sgDAD1 NPs in penetrable tumor lethality. PLL is biodegradable and has been approved as a food antibacterial agent by the Food and Drug Administration (FDA), indicating its biosecurity 35. Moreover, a safe dose of PLLF was used to deliver the Cas9-sgDAD1 plasmid. Thus, bladder tumor inhibition by PLLF/Cas9-sgDAD1 NPs was attributed to the efficient knockout of DAD1. The underlying mechanisms of tumor cell apoptosis caused by PLLF/Cas9-sgDAD1 NPs were explored by performing RNA-seq analysis, comparing differentially expressed genes (DEGs) between PLLF/Cas9-sgDAD1 NP-treated and untreated cells. A total of 33 DEGs were identified **(Figure S11)**. The Kyoto Encyclopedia of Genes and Genomes (KEGG) analysis of these DEGs revealed several dominant pathways, such as the TNF and MAPK pathways, associated with the proliferation, differentiation, and apoptosis of cancer cells (**Figure 5G**). The activities of ERK, P38, and JNK were also examined using western blotting because of their crucial roles as signal transducers in the MAPK pathway. The decreased expression of p-ERK, p-P38, and p-JNK shown in **Figure 5H** revealed that PLLF/Cas9-sgDAD1 NPs induced tumor cell apoptosis by inhibiting the ERK signaling pathway. The mechanism underlying the apoptosis induced by the knockout of DAD1 remained unclear. The well-known apoptosis-regulating gene Bcl-2 could not reverse the apoptosis caused by DAD1 deletion, indicating that the deletion of DAD1 triggered apoptosis through the endoplasmic reticulum (ER) pathway rather than through the death receptor and mitochondrial pathways 47. In addition, the upregulation of DAD1 contributes to cisplatin resistance in cancer cells, and cisplatin is an essential chemotherapeutic drug for treating BCa 52. For the first time, the MAPK signaling pathway was found to be involved in apoptosis induced by DAD1 deletion, providing a promising new target for BCa treatment.

### Intravesical anticancer evaluation of PLLF/Cas9-sgDAD1 NPs

**Figure 6:**
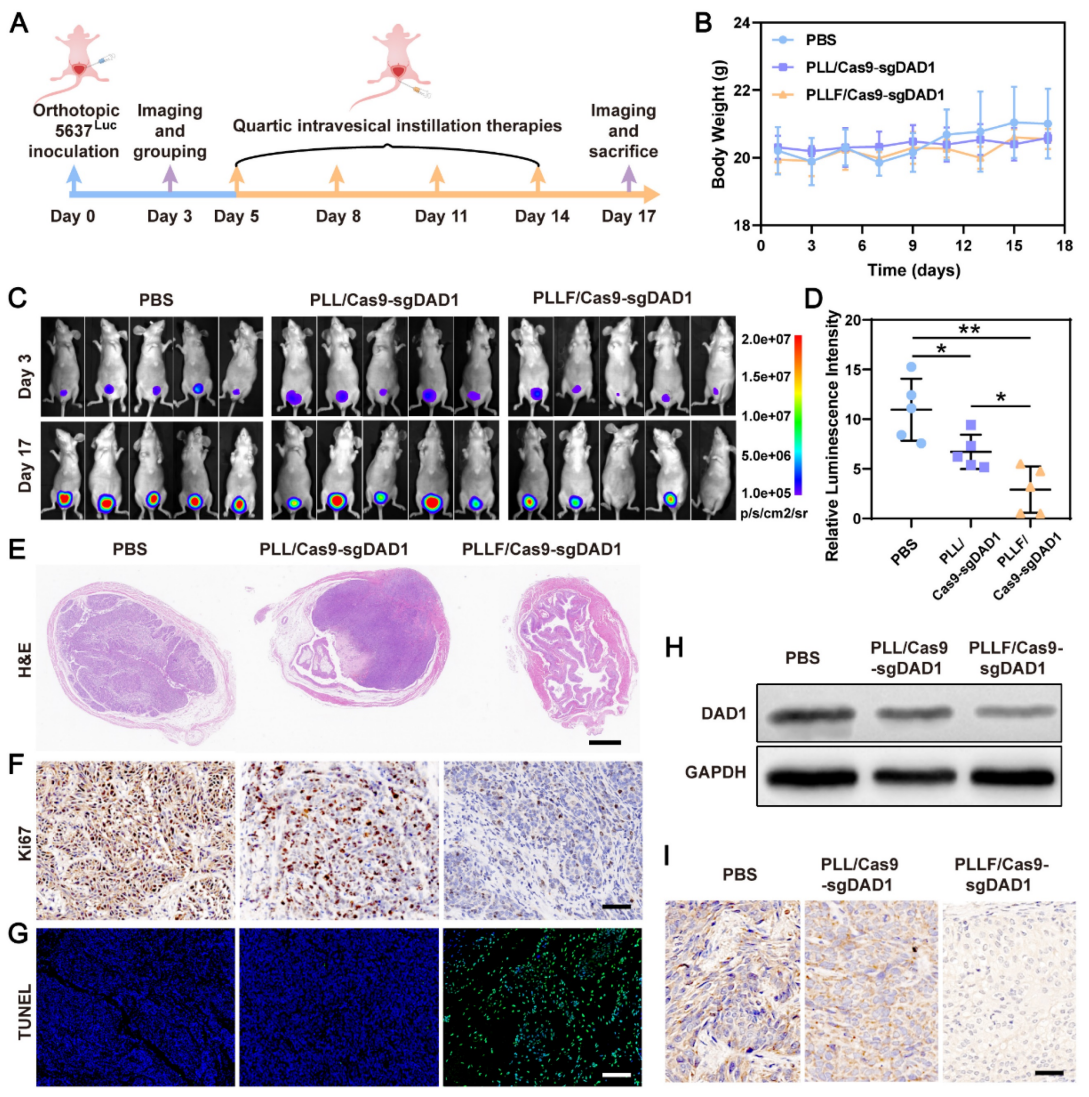
** Intravesical anticancer evaluation of PLLF/Cas9-sgDAD1** NPs**. A)** Schematic presentation of intravesical instillation therapy for orthotopic bladder tumor using PLLF/Cas9-sgDAD1 NPs. **B)** Body weights of the involved tumor-bearing nude mice in PBS, PLL/Cas9-sgDAD1, PLLF/Cas9-sgDAD1 groups during treatment course. **C)** The *in vivo* bioluminescence images of tumor-bearing nude mice with treatment of PBS, PLL/Cas9-sgDAD1, and PLLF/Cas9-sgDAD1 on Day 3 and Day 17. **D)** Relative quantitative analysis of bioluminescence signals (the ratio of Day 17 and Day 3) from different groups. **E)** H&E-stained sections of the representative bladders resected from nude mice with different treatments. Scale bars: 1 mm. **F)** Ki67 staining (brown) and **G)** fluorescence TUNEL staining (green) images of tumor sections from different treatment groups. Scale bars: 50 μm. **H)** Western blotting and** I)** immunohistochemistry analysis of DAD1 expression in tumor tissues from different treatment groups. Scale bars: 50 μm. Statistic significances were calculated by the Student's *t*-test two tailed. *P < 0.05, **P < 0.01.

Based on the favorable *in vitro* results confirming the specific inhibition of bladder tumor growth and the excellent mucosal penetrability of PLLF/Cas9-sgDAD1 NPs, intravesical therapeutic experiments were performed using a bladder orthotopic tumor mouse model. To establish this mouse model, 5×10^5^ 5637 cells stably expressing firefly luciferase, denoted as 5637^Luc^, were inoculated into the bladder walls of female BALB/c nude mice (6-8 weeks old). Tumor establishment was confirmed by the presence of bioluminescence on day 3, and then tumor-bearing mice were randomly divided into three groups, with five mice in each group, for treatment with different formulations, including PBS, PLL/Cas9-sgDAD1, and PLLF/Cas9-sgDAD1. As illustrated in **Figure 6A**, intravesical instillation therapy was implemented every three days for a total of four times, and tumor growth was again observed using the IVIS Lumina system for bioluminescence imaging on day 17. The body weights of all nude mice were monitored throughout the course of administration. The tolerable fluctuation in the curves depicting body weight shown in **Figure 6B** demonstrates the negligible systemic toxicity of PLL/Cas9-sgDAD1 and PLLF/Cas9-sgDAD1 via intravesical administration. A comparison of the bioluminescence intensity of orthotopic bladder tumors at the start and end (days 3 and 17, respectively) revealed the anticancer efficacy of PLLF/Cas9-sgDAD1 NPs (**Figure 6C**).

Statistical analysis of relative bioluminescence intensity (the ratio of day 17 to day 3) showed a significant impediment of tumor growth in the PLLF/Cas9-sgDAD1 treated group compared to the PBS-or PLL/Cas9-sgDAD1 treated group (**Figure 6D**). The mice were then dissected, and the whole bladder and major organs, including the heart, liver, spleen, lungs, and kidneys, were resected for further analysis. Among these organs, only the bladder excised from the PBS- and PLL/Cas9-sgDAD1-treated groups showed bioluminescence signals (**Figure S12**), indicating no tumor metastasis in the therapeutic process. The bladder excised from PLLF/Cas9-sgDAD1 treated mice was visually smaller and softer to touch than those from other groups (**Figure S13A**). The bladder weight of the PLLF/Cas9-sgDAD1-treated group was markedly lower than that of the other two groups (**Figure S13B**). To further observe the orthotopic bladder tumor, the entire excised bladder was sectioned and stained using H&E staining analysis. The images shown in **Figure 6E** demonstrate a stuffed solid tumor in the bladder of the PBS- or PLL/Cas9-sgDAD1-treated mice, whereas tumor residue was scarce in the bladder of the PLLF/Cas9-sgDAD1 treated mice. In addition, Ki67 and TUNEL histological analyses were conducted to evaluate anticancer activity. The cell proliferation marker Ki67 stained brown and had an extremely impaired expression level in the tumor tissue of the PLLF/Cas9-sgDAD 1 group (**Figure 6F**). Increased green fluorescence signals representing apoptotic cells were observed in the tumor tissue of the PLLF/Cas9-sgDAD1 group (**Figure 6G**).

To clarify the tumoricidal mechanism of PLLF/Cas9-sgDAD1 NPs, DAD1 expression in orthotopic bladder tumor cells was analyzed. Western blotting and immunohistochemistry demonstrated the downregulation of DAD1 expression after treatment with PLLF/Cas9-sgDAD1 NPs (**Figure 6H-I**), indicating the successful delivery of the Cas9-sgDAD1 plasmid by the PLLF nanomicelles. Moreover, the biosafety of PLLF/Cas9-sgDAD1 NPs after intravesical instillation was assessed by H&E staining of major organs. Based on the images of the H&E-stained sections (**Figure S14**), no apparent pathological alterations were caused by repeated intravesical administration of PLLF/Cas9-sgDAD1 NPs. To explore the final fate of PLLF after intravesical instillation, PLL and PLLF were labeled with Cy5.5 to form PLL_cy5.5_/Cas9-sgDAD1 and PLLF_cy5.5_/Cas9-sgDAD1 NPs before intravesical administration. Then we observed the distribution of Cy5.5 fluorescence in mouse using an IVIS Lumina system at 24 h, 72 h, 120 h and 168 h respectively. As shown in **Figure S15**, a quite proportion of PLL or PLLF NPs could be excreted with urine within 24 hours, and the residues were detained in bladder without transferring into other organs significantly, which were finally metabolized over time. It's interesting that PLLF NPs were capable of detaining in bladder with a longer time than that of PLL NPs, which may attribute to the good transmucosal ability of PLLF NPs. These results confirm the superior performance of PLLF for tissue penetration and delivery of a large CRISPR-Cas9 plasmid *in vivo*. In addition to the well-known “fluorous effect”, the bio-inertness of fluoropolymers is also crucial, where PLLF/Cas9-sgDAD1 NPs experience insignificant disturbances from biocomponents *in vivo* 29. The excellent antitumor capability of the PLLF/Cas9-sgDAD1 NPs also provides valid evidence for their clinical application in BCa therapy. This evidence generally indicates the biosafety and reliable performance of PLLF/Cas9-sgDAD1 NPs in eradicating orthotopic bladder tumors with favorable biocompatibility.

## Conclusion

In summary, an effective intravesical instillation system capable of delivering therapeutic CRISPR-Cas9 plasmids into BCa cells by penetrating the bladder mucosa was developed, resulting in satisfactory therapeutic outcomes in *in vitro* cell experiments and *in vivo* in orthotopic BCa mouse models. Through the fluorination modification of PLL, PLLF served as carriers to deliver therapeutic CRISPR-Cas9 plasmids for intravesical instillation therapy of BCa were successfully synthesized. PLLF readily adsorbed plasmids via electrostatic interactions, forming stable and compact NPs that exhibited superior plasmid transfection, cross-membrane and transmucosal penetration capacities. In addition, the glycosylation modification-associated gene DAD1 had a relatively high expression level in BCa tissues and significantly affected the prognosis of patients with BCa. Furthermore, PLLF/Cas9-sgDAD1 NPs have been demonstrated to extend the detaining time in bladder and suppress orthotopic bladder tumors via intravesical instillation effectively with favorable biocompatibility. This work presents an innovative intravesical instillation strategy for gene therapy in BCa cells with high potential for clinical application.

## Supplementary Material

Supplementary figures.
