# Supplementary material for: Targeting DAD1 gene with CRISPR-Cas9 system transmucosally delivered by fluorinated polylysine nanoparticles for bladder cancer intravesical gene therapy
Source: Theranostics. 2024 Jan 1;14(1):203–19. doi: 10.7150/thno.88550 (PMC10750211; doi:10.7150/thno.88550)
Supplement: Supplementary file 1 — Supplementary figures. [file thnov14p0203s1.pdf]

Supporting Information

Targeting DAD1 gene with CRISPR-Cas9 system transmucosally delivered by fluorinated polylysine nanoparticles for bladder cancer intravesical gene therapy

Dongdong Tang[1,2,4#], Yang Yan[2,5#], Yangyang Li[2#], Yuqing Li[3], Junqiang Tian[1], Li Yang[1], Hui Ding[1], Ghassan Bashir[3], Houhong Zhou[3], Qiuxia Ding[2], Ran Tao[2], Shaohua Zhang[2,3], Zhiping Wang[1]* Song Wu[1,2,3]**

1. Department of Urology, Lanzhou University Second Hospital, Lanzhou 730030, P.R. China

2. Department of Urology, The Third Affiliated Hospital of Shenzhen University (Luohu Hospital Group), Shenzhen University, Shenzhen 518000, China

3. Department of Urology, South China Hospital, Medical School, Shenzhen University, Shenzhen 518000, China

4. Department of Urology, The First Affiliated Hospital of Zhengzhou University, Zhengzhou, 450052, P. R. China

5. Songshan Lake Materials Laboratory, Dongguan, 523808, P. R. China

*Correspondence: wangzplzu@163.com; wusong@szu.edu.cn; 22zsh@163.com

[#]These authors contributed equally to this work.

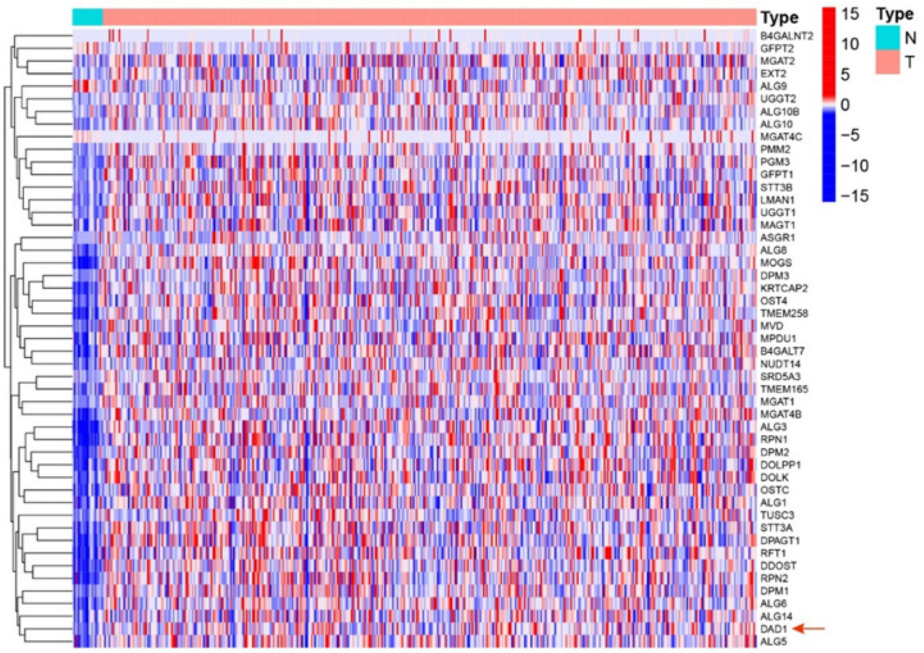

Figure S1 The heatmap of 49 glycosylation modification differential genes in bladder cancer. The red arrow indicates the target gene DAD1.

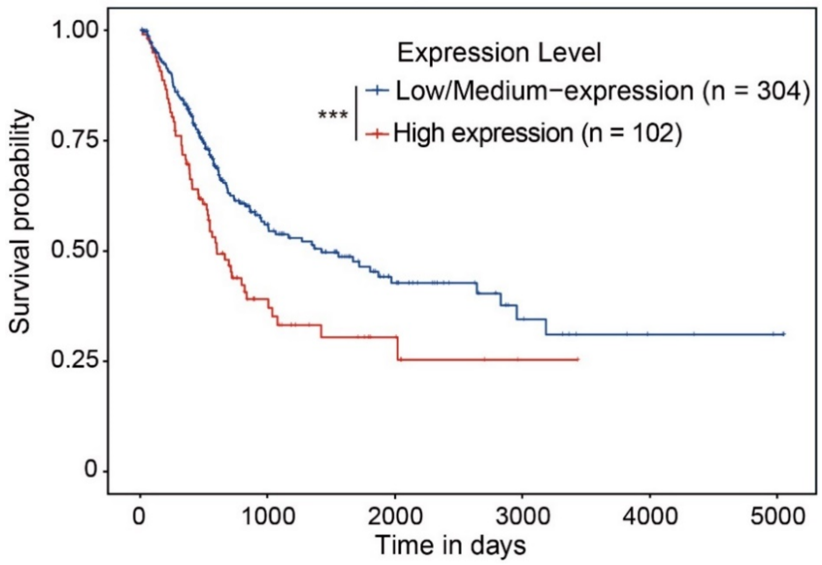

Figure S2 Kaplan- Meier survival curve of DAD1 high expression and low expression groups acquired by using UALCAN (http://ualcan.path.uab.edu/analysis.html) based on TCGA bladder cancer cohort. ***P < 0.001.

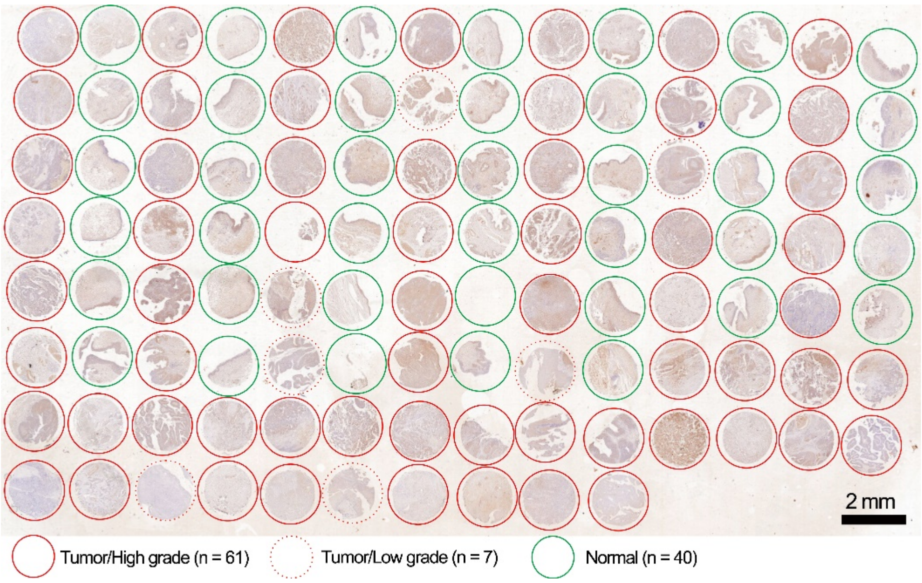

Figure S3 The IHC staining of DAD1 in human tissue microarray HBlaU108Su01. The red solid line indicates the high-grade bladder tumor. The red dotted line represents the low-grade bladder tumor.

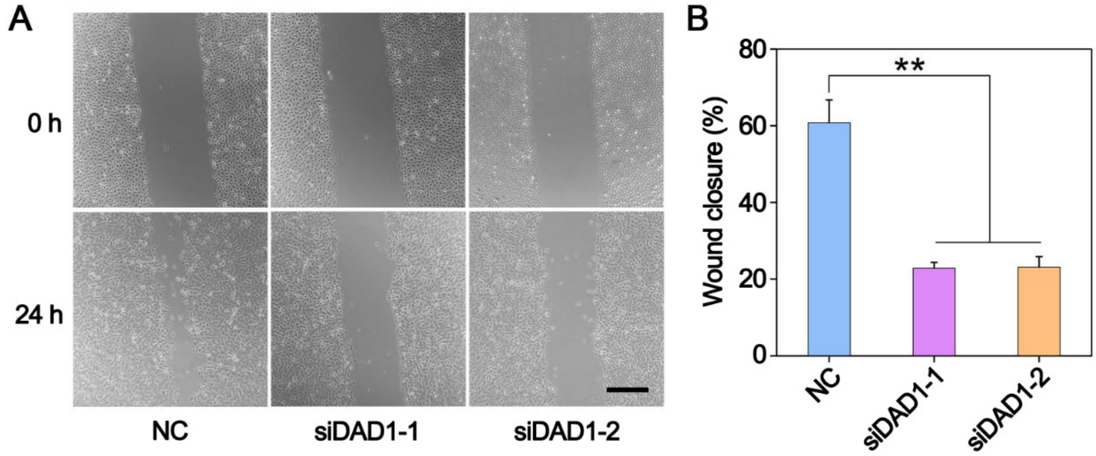

Figure S4 The representing images of wound healing after knockdown of DAD1 in 5637 cells by siRNAs. **P < 0.01. Scale bars: 100 μm.

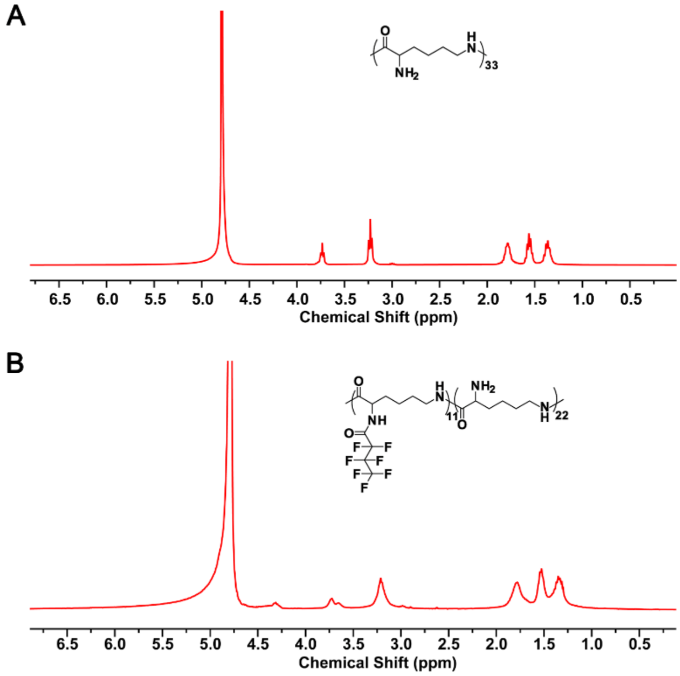

Figure S5 ^{1}H NMR spectra of PLL and PLLF.

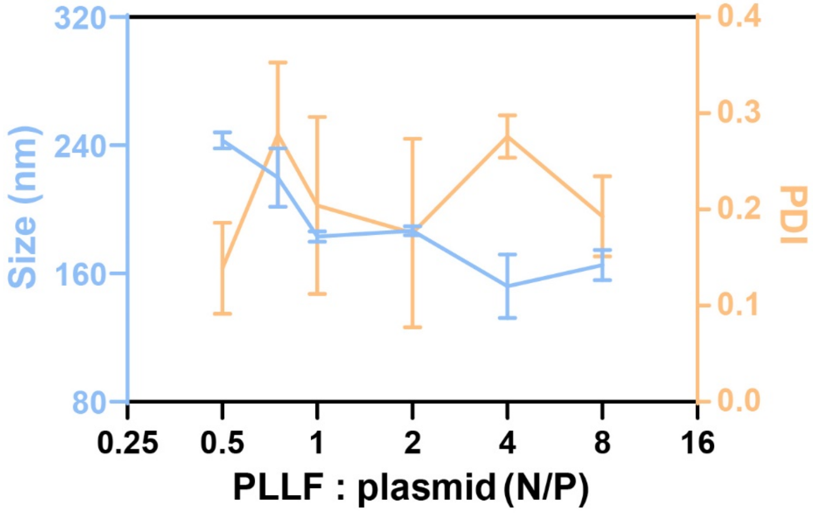

Figure S6 The size and polymer dispersity index (PDI) of PLLF/CRISPR-Cas9 NPs at different N/P ratios.

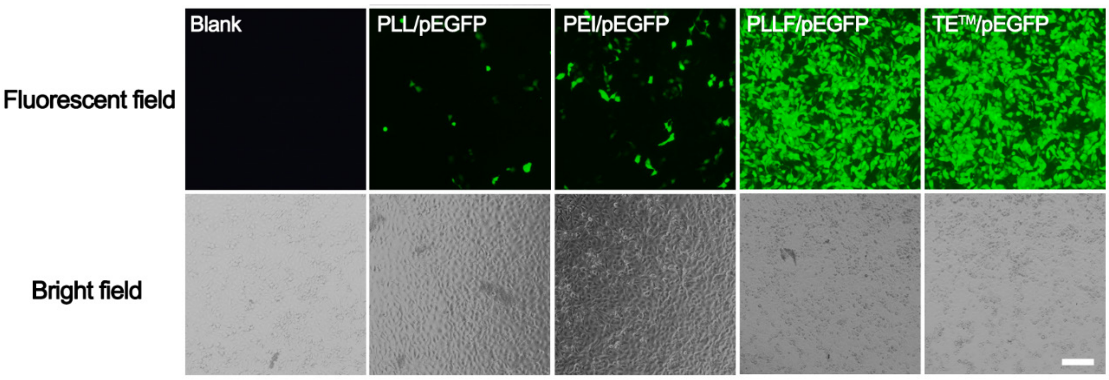

Figure S7 Fluorescence and bright field images of EGFP expression in 5637 cells after pEGFP plasmids were transfected with PLL, PEI, PLLF and TETM at 48h. Scale bars: 100 μm.

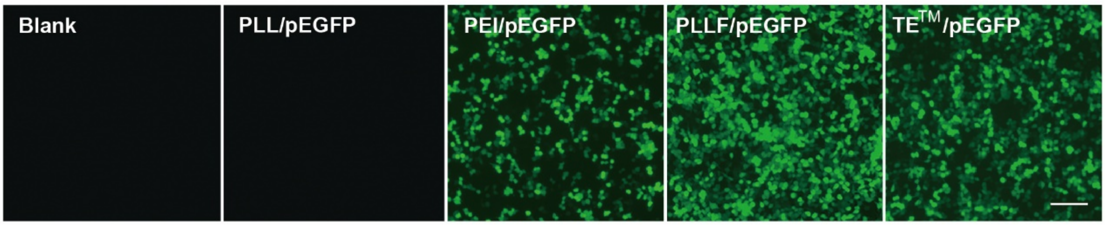

Figure S8 Fluorescence images of EGFP expression in human embryonic kidney cell line 293T after pEGFP plasmids were transfected with PLL, PEI, PLLF and TE^{TM}. Scale bars: 100 μm.

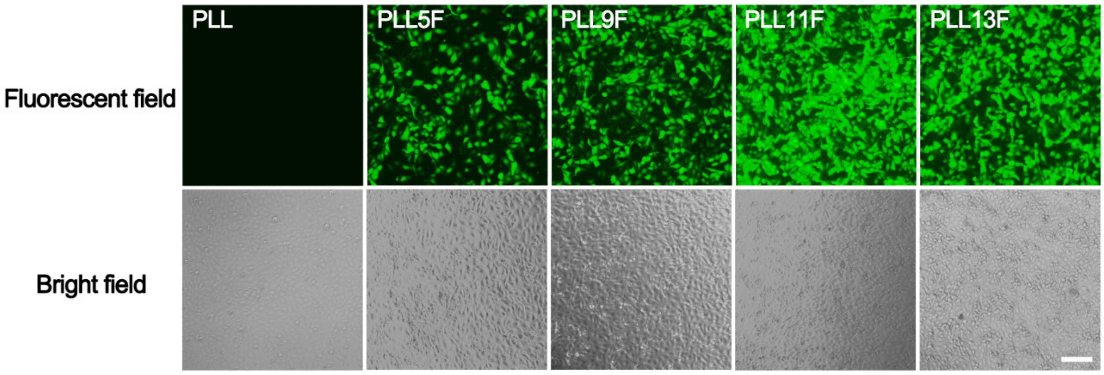

Figure S9 EGFP plasmid transfection in 5637 cells at 48 h by the unmodified PLL and the different PLLFs. Scale bars: 100 μm.

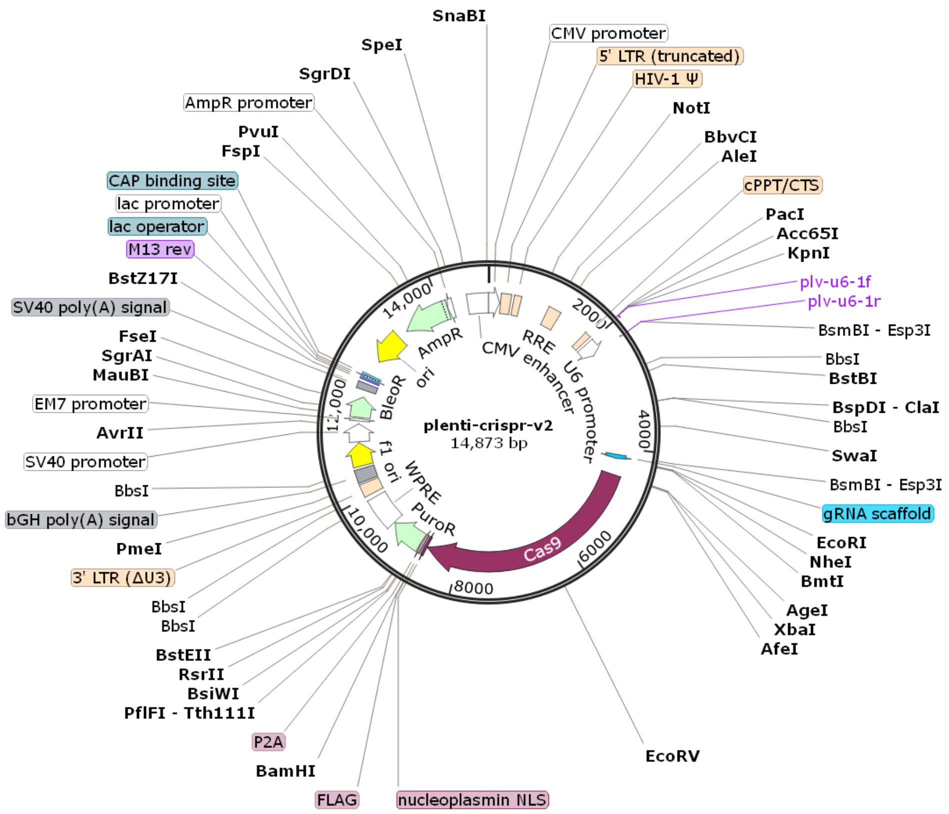

Figure S10 The plasmid map used to construct the Cas9-sgDAD1 plasmid. The blue sequences were sgDAD1 integrated in the plasmid.

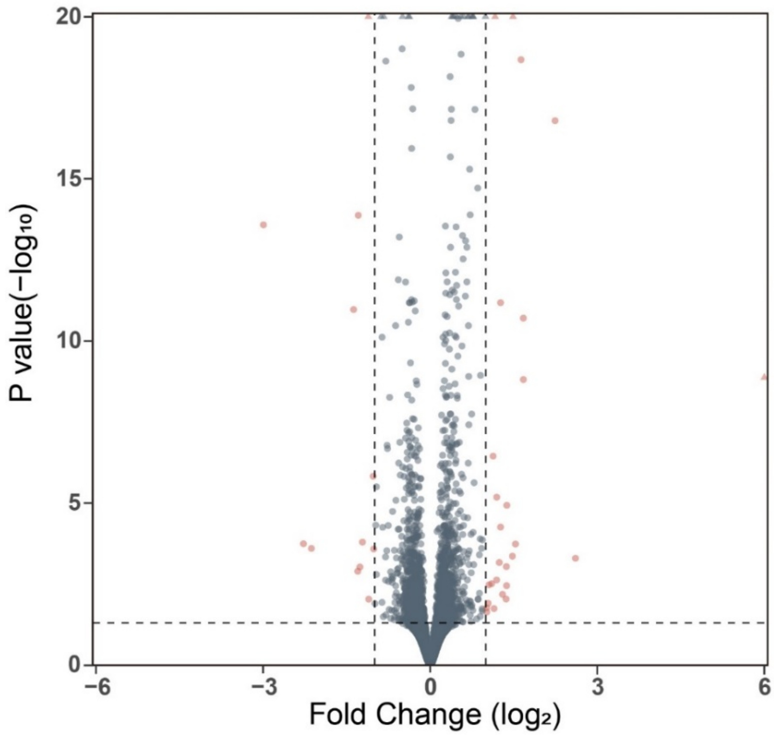

Figure S11 The volcano map of 33 different expression genes after treatment of PLLF/Cas9-sgDAD1 nanoparticles. The red dots represent the genes met the criterion $|Log_2(\text{fold change})| > 1$ and p-value < 0.05.

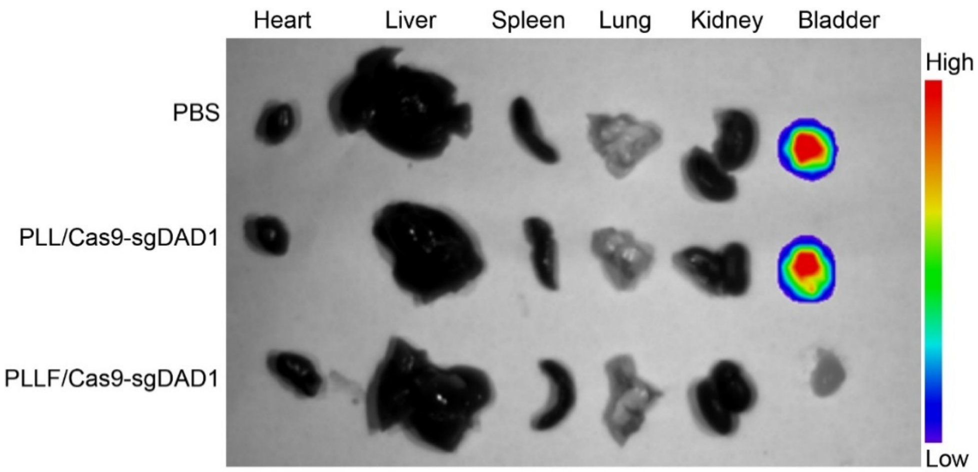

Figure S12 The bioluminescence signal of heart, liver, spleen, lung, kidney, and bladder in PBS, PLL/Cas9-sgDAD1 and PLLF/Cas9-sgDAD1 groups.

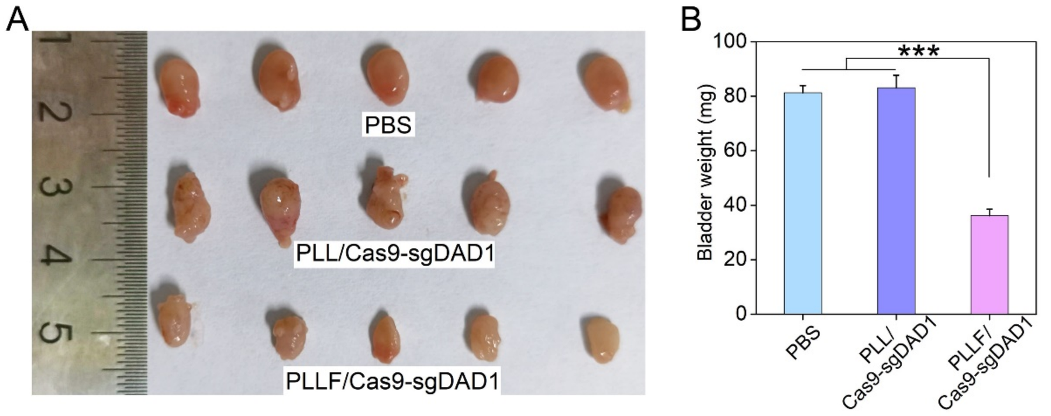

Figure S13 The images (A) and weight (B) of bladders in PBS, PLL/Cas9-sgDAD1 and PLLF/Cas9-sgDAD1 groups. ***P < 0.001.

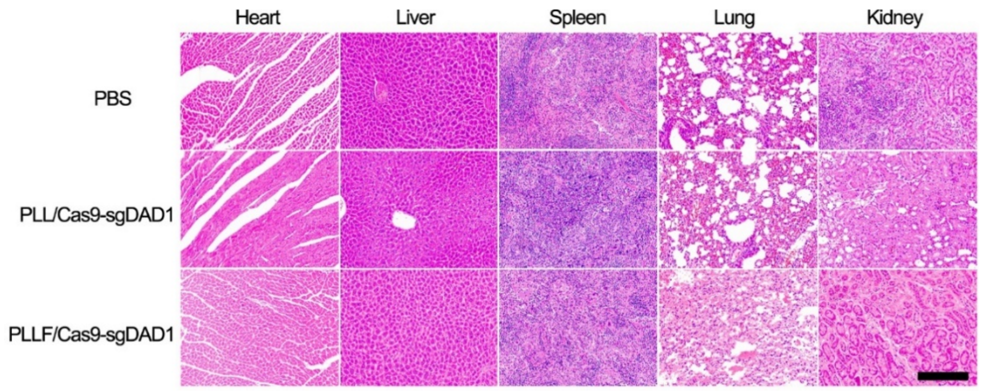

Figure S14 The H&E staining of major organs including heart, liver, spleen, lung, kidney in PBS, PLL/Cas9-sgDAD1 and PLLF/Cas9-sgDAD1 groups. Scale bars: 200 μm.

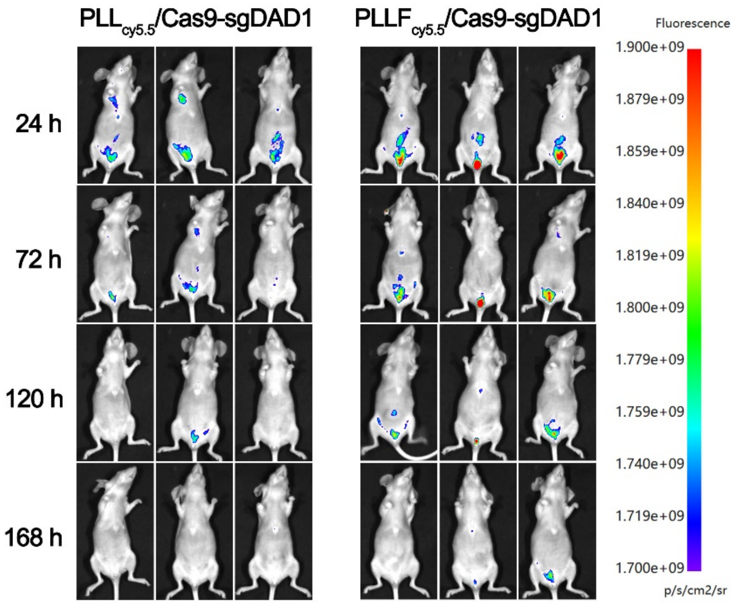

Figure S15 Fluorescence images of mice after intravesical instillation of $PLL_{cy5.5}$/Cas9-sgDAD1 and $PLLF_{cy5.5}$/Cas9-sgDAD1 nanoparticles at different time intervals.
